# Coding Efficiency of Fly Motion Processing Is Set by Firing Rate, Not Firing Precision

**DOI:** 10.1371/journal.pcbi.1000860

**Published:** 2010-07-22

**Authors:** Deusdedit Lineu Spavieri, Hubert Eichner, Alexander Borst

**Affiliations:** Department of Systems and Computational Neurobiology, Max-Planck-Institute of Neurobiology, Martinsried, Germany; University College London, United Kingdom

## Abstract

To comprehend the principles underlying sensory information processing, it is important to understand how the nervous system deals with various sources of perturbation. Here, we analyze how the representation of motion information in the fly's nervous system changes with temperature and luminance. Although these two environmental variables have a considerable impact on the fly's nervous system, they do not impede the fly to behave suitably over a wide range of conditions. We recorded responses from a motion-sensitive neuron, the H1-cell, to a time-varying stimulus at many different combinations of temperature and luminance. We found that the mean firing rate, but not firing precision, changes with temperature, while both were affected by mean luminance. Because we also found that information rate and coding efficiency are mainly set by the mean firing rate, our results suggest that, in the face of environmental perturbations, the coding efficiency is improved by an increase in the mean firing rate, rather than by an increased firing precision.

## Author Summary

How is information about the sensory world represented in the brain? How does this representation change, when the stimulus is contaminated by noise or the brain itself is perturbed by temperature variations? We address these questions by studying motion vision in the fly's visual system. Flies barely thermoregulate but behave normally within a large temperature range. Moreover, visual stimuli are naturally noisy at low intensities and might change considerably within short periods of time. To analyze how the fly's brain handles such perturbations, we recorded responses of a motion-sensitive neuron at different temperatures and light intensities. We analyzed how the responses change and how much information about the stimulus is lost due to noise. We found that response variability and delay are determined mainly by the light intensity, but are barely affected by temperature variations. The information content of the responses was weakly influenced by response noise or delay. The mean firing rate of the neuron, which was strongly influenced by temperature and light level, determined how much stimulus information was contained in response. Based on our results, we can say that over a wide range of environmental conditions, their influence on the efficiency of the system to process information is small.

## Introduction

Several flying insects are able to partially control the temperature of (part of) their bodies during certain periods of time, by means of physiological or behavioral strategies [1]–[3]. Blowflies (*Calliphora vicina*) however, barely thermoregulate. During flight, when considerable amount of heat is produced by the flight muscles, the temperature of their thorax increases by about five degrees Celsius. The head temperature increases less - by just about two degrees above the temperature of environment in the same period of time. Head and thorax temperature reach the environment temperature around one hundred seconds after the flight stops [4]. Therefore, their body temperature closely follows the temperature of the environment, which can easily vary more than ten degrees during the day. Within this range, the kinetics of biochemical reactions underlying neural information transmission might vary between three and tenfold [5], and indeed, a strong impact on several response properties of fly's photoreceptors [6], [7] and other interneurons [8], [9] has been observed. Despite the effect of temperature on their nervous system, blowflies show normal flight behavior over a wide temperature range (10–37°C) [10], [11].

One of the processes influenced by temperature changes is the adaptation of the photoreceptors to light intensity variations. Adaptation to light intensity is necessary to match the limited dynamic range of the photoreceptor responses to encode the wide range of intensities found in natural conditions. Moreover, at low light intensities, the combination of the discrete nature of light and the high sensitivity of the photoreceptors introduces noise into the system, which seems to be responsible for around 50% of the noise measured in the response of postsynaptic cells [12]. The consequences of this noise further downstream the visual pathway are still being debated [13]–[17].

**Figure 1:**
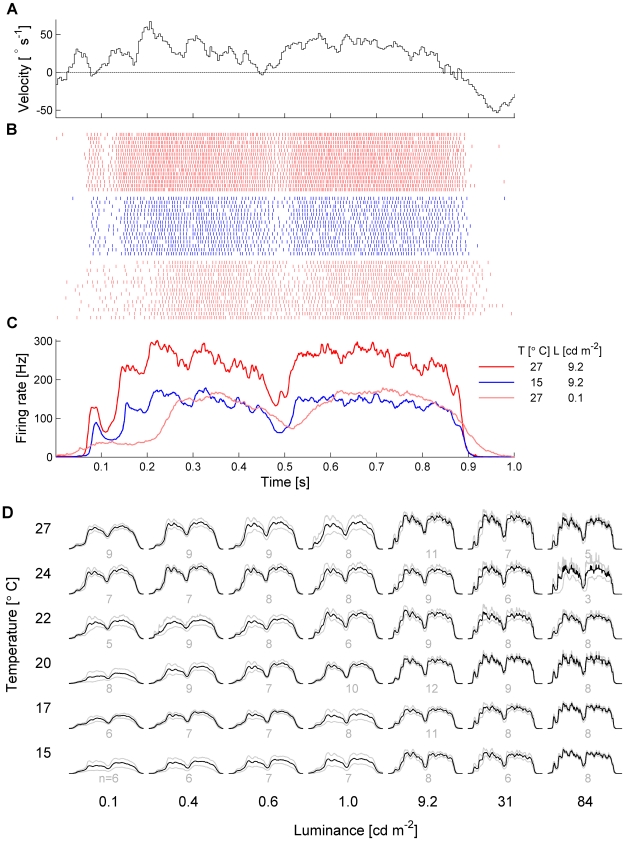
Time dependent-firing rate. **A** A segment of the image velocity as a function of time. **B** Raster plots of the same H1 at different experimental conditions of temperature and light intensity. **C** Respective average rates calculated using 137 trials for each condition. **D** Average rates of 325 acquisitions (84 flies) in a wider set of experimental conditions. Scales are the same as indicated in **C**. For each condition, firing rates of several flies were pooled - n is the sample size and the gray curves are bootstrap confidence intervals (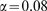, 1000 replications).

Here, we determine to what extent temperature and luminance influence the firing rate and precision of the responses of an identified wide-field, motion-sensitive neuron in the fly's visual system called ‘H1-cell’ [18], and how the response properties of H1 contribute to information transmission in the system. The H1-cell is located in the posterior part of the third neuropil of the fly's visual system, the so-called lobula plate [19], [20]. It is excited by ipsilateral horizontal back-to-front motion, and inhibited by motion in the opposite direction, i.e. front-to-back. H1 is part of a network of about 60 neurons [21]–[25] that supply the neck and flight motor system with motion information for flight stabilization and course control [26]–[31]. We recorded H1 responses from 84 flies to a square wave grating moving with a time-varying velocity (Fig. 1A) at 42 different combinations of temperature and luminance. The temperature range covered about half of the range flies face under natural conditions. The luminance range covered four orders of magnitude and includes intensities where photon noise effects are common, corresponding to dusk and dawn under natural conditions.

## Results/Discussion

### Experimental Findings

The effects of temperature and luminance on H1 responses are dramatic, but strikingly different for the two parameters (Fig. 1B, C). Under warm and bright conditions (red spikes in Fig. 1B, red line in Fig. 1C), the cell's firing activity follows the time course of the stimulus velocity with a short delay, reaching maximum firing frequencies of about 300 Hz for velocities along the preferred direction of the cell. Compared to that, a temperature reduction decreases the mean firing rate but leaves the temporal structure of the response unaffected (blue spikes in Fig. 1B, blue line in Fig. 1C). In contrast, a luminance reduction reduces not only the mean firing rate, but additionally alters the temporal structure of the response (light red spikes in Fig. 1B, light red line in Fig. 1C). The full spectrum of responses for all parameter combinations is shown in Fig. 1D.

In order to quantify the effect of temperature and luminance on the various response properties like firing rate and reliability, we calculated for each luminance a temperature coefficient Q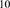 (the ratio of the respective response parameter at 25

C and 15

**Figure 2:**
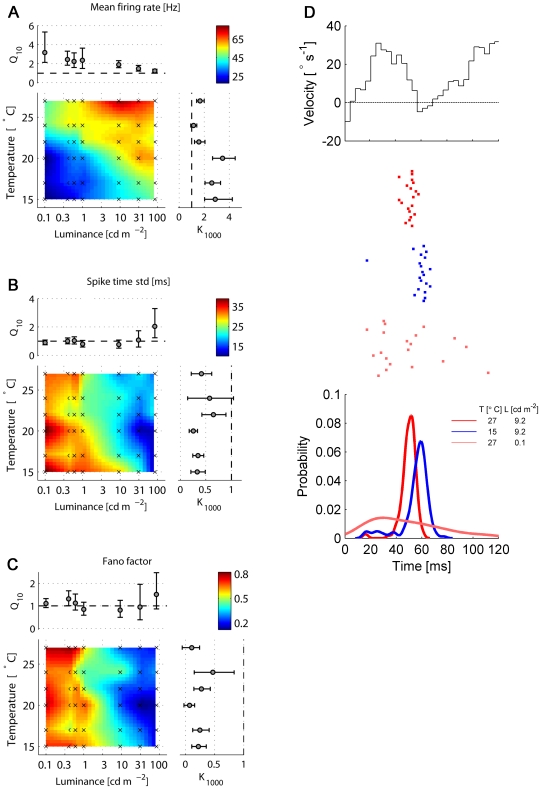
Firing rate and precision as a function of temperature and light intensity. **A** Mean firing rate. **B** Spike jitter, measured as the standard deviation of the first spike after a velocity transient (from negative to positive). **C** Average Fano factors for transient responses 50 ms after a velocity transition, calculated in an interval of 50ms, using overlapping windows of 20 ms. Color code represents the linear interpolation of the mean values at the experimental points, indicated by the crosses. Q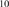 and K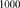 are the temperature and luminance coefficients, respectively (for details see text). Error bars are bootstrap confidence intervals (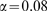, 1000 replications) **D** Top: Segment of a stimulus waveform during a velocity transition from inhibitory to excitatory direction. Center: Raster plots of the responses in 20 trials at three different conditions, indicated in the legends. Each mark represents the occurrence of one spike. Bottom: Distributions of the arrival time of the first spike for the three different conditions shown above, obtained using a kernel density estimator with optimized width for Gaussian distributions.

C). A strong influence of temperature expresses itself in a Q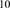 value different from 1 at many different luminance levels. Furthermore, we calculated at each temperature a luminance coefficient, K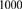, as the ratio of the response parameter at 100 cd m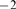 and at 0.1 cd m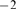. Again, a strong influence of luminance on a given response property is revealed by K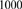 values different from 1 at many different temperatures. Both temperature and luminance have a strong effect on the mean firing rate of H1, with Q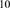 and K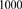 values of up to 3 (Fig. 2A). However, temperature has almost no effect on the response reliability while luminance does (Fig. 2 B–D). We quantified the response reliability in two different ways: first, we measured the standard deviation (STD) of the occurrence times of the first spike after a velocity transition, i.e. a zero-crossing from an inhibitory to an excitatory direction of motion (Fig. 2D). This measure should, at least in principle, be independent of the mean firing rate. Second, we measured the response reliability 50 ms after a velocity transition using the ratio of the variance of the spike count and the mean spike count (Fano Factor) within a 20 ms time window (Fig. 2C). Here, a better response reliability can be achieved in two ways - a smaller variance or a higher firing rate associated with the refractory period of the neuron [32]. The effect of temperature on the STD or on the Fano Factor is rather small: at almost all light intensities tested, the coefficients of temperature (Q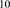) are not statistically different from 1 (Fig. 2B,C). In contrast, the reliability of the response improves considerably for all measures of reliability (all p
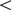
0.001, Wilcoxon test) with increasing luminance. The coefficient of luminance (K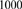) reaches values smaller than 0.5 at several temperatures measured. These findings are summarized by the Spearman correlation coefficients between response properties and temperature and luminance (Table 1). In summary, we find that temperature does not significantly affect the response reliability, while higher luminance values increase the response reliability substantially (Figs. 2 B,C).

**Table 1:** Correlation between response properties and temperature and light intensity.

Response properties	Temperature	Light intensity
Mean firing rate	0.47	0.44
Fano factor (transient)	0.04 ( 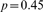 )	−0.64
STD first spike	−0.005 ( 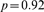 )	−0.55

Spearman rank correlation. The p values not shown were smaller than 0.05 (n = 325).

It is interesting to note that neither the increase of photoreceptor bandwidth [7] nor the temperature dependent spontaneous activity of the system has a strong impact on the reliability of the responses. However, the effect of temperature on the firing rate depends on the mean luminance - the Q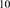 coefficients decrease with increasing luminance (Fig. 2A). We observed similar interdependent effects of temperature and luminance in the spontaneous firing rate of H1: The Q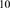 value in the dark was around 6.4 and fell to 3.3 in the presence of a stationary image with a luminance of 84 cd m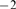 (n = 5 flies). This effect has also been measured in several response properties of the fly's photoreceptors [6], [7] such as e.g. the bandwidth, which has a Q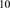 of 3 when the cells are dark adapted and 1.9 when they are light adapted [7]. What consequences will such effects have on the amount of information about the stimulus in the H1 response? To what extent do the firing rate and firing precision determine the efficiency of the system to encode information?

### Information Theoretic Analysis

To answer these questions, we estimated the information rate and coding efficiency of H1 responses. To calculate these quantities, we discretized the response in bins of 2ms and represented the occurrence of a spike in a bin by ‘1’ and the absence by ‘0’. Probability distributions of binary words with lengths from 2ms up to 20 ms were estimated and information rates calculated. The temporal scale of the system (encoding window) was defined as the length of the binary words at which the information rate was maximal (see Materials and Methods, and Fig. S1 for details). The behaviorally relevant temporal scales for flies, estimated from flight trajectories in which males flies pursued females, are about 40ms [33]. This is the total delay of the system, from the detection of the female in the visual field up to the correction of the flight course. Therefore, it is reasonable to expect that the information transmission in the system occurs in shorter time-scales. Moreover, changes in the temporal scale of the response are known to be part of the mechanisms of adaptation - e.g., temperature and luminance alter the response time-scales of the photoreceptors [7] - and should thus be taken into account. We also analyzed the importance of the precise spike timing on the information rate by using two encoding modes to determine it: one which recognizes the exact positions of the spikes within the encoding window (‘timing’) to discriminate responses and one that just counts the number of spikes within it (‘count’).

In a similar way as the encoding window, the latency of the system was defined as the lag between the velocity and H1 responses that maximizes the mutual information between them. To calculate the mutual information, we estimate the joint probability distribution between binary response words and instantaneous velocity, both discretized in bins of 2ms. The velocity amplitude was discretized in bins of 1 degree per second and the length of the word used was the one that maximizes the information rate between stimulus and response (see Materials and Methods, and Fig. S1 for details).

**Figure 3:**
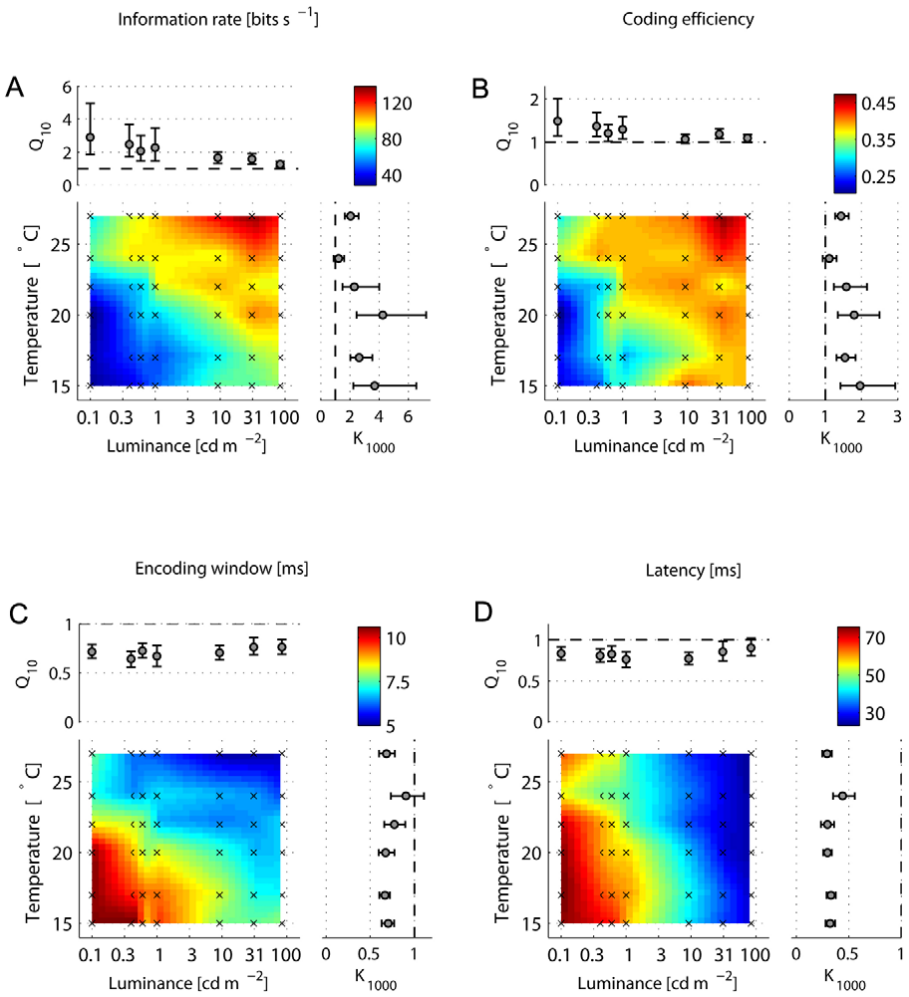
Information rates, efficiencies and optimal time-scales as a function of temperature and luminance. **A**. Optimal information rates and **B**. respective coding efficiencies. **C**. Optimal encoding windows and **D**. latencies. Color code represents the linear interpolation of the mean values at the experimental points, indicated by the crosses. Q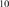 and K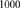 are the temperature and luminance coefficients, respectively (for details see text). Error bars are bootstrap confidence intervals (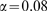, 1000 replications).

The dependence of the information rate on luminance and temperature is similar to the dependence of the mean firing rate (Figs. 2A and 3A). Here again, temperature and luminance have interdependent effects on the information rate. The temporal precision of responses after velocity transitions (Fig. 2B,D) is set mainly by the mean light level and does not have a great impact on the information rate, as previous work suggested [14]: as temperature increases, the information rate increases too (Fig. 3A), although the firing precision remains the same. Indeed, the partial correlation coefficient between the information rate and the STD of the first spike, with the effect of the mean firing rate removed, is 0.23, whereas the correlation with the mean firing rate, with the effect of the STD removed, is 0.91 (Table 2). Thus, the information rate is determined mostly by the mean firing rate, rather than by the noise in the system or in the input signal. The information rate of the spike-timing mode at an optimal encoding window was on average only 1.06 bits s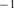 (n = 325) higher than of the count encoding mode.

**Table 2:** Correlation between fundamental and information theoretic response properties.

Response property	Rate	Rate 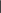 Precision	Precision	Precision 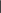 Rate
Information rate	0.94	0.91	−0.58	0.23
Coding efficiency	0.72	0.68	−0.37	0.23
Encoding window	−0.83	−0.86	0.36	−0.51
Latency	−0.71	−0.45	0.69	0.40

Spearman rank correlation. Firing precision is the standard deviation of the first spike after a velocity transition. All p values were smaller than 0.01 (n = 325).

The coding efficiency is more robust against changes on temperature or luminance than the information rate (Fig. 3B). Again, the effect of thermal fluctuations is reduced as the luminance increases. Observing the partial correlation coefficients (Table 2), we can see the temporal precision of the response does not contribute as much as the mean firing rate to the coding efficiency. Moreover, the sign of the correlation coefficient between coding efficiency and firing precision is reversed when the effect of the mean firing is removed. This means that the firing rate masked the real effect of the firing precision on the coding efficiency, mainly because both firing rate and precision vary when the light intensity changes. The count encoding mode was on average 15% more efficient to convey information than the timing encoding mode at the respective optimal encoding windows. These results suggest that the improvement of the information rate and coding efficiency is achieved by an increase of the mean firing rate, rather than the firing precision.

Why does the fly additionally increase the firing precision as the mean light level increases, if the efficiency of the system to convey information barely changes? The amount of information transmitted is not the only essential signal characteristic needed for survival - the temporal scale of the system is also fundamental. Here we estimated two response temporal scales - the optimal encoding window and the latency (see Materials and Methods, and Fig. S1). The encoding window decreases with luminance (p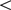 0.001) and temperature (p
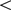
0.001) (Fig. 3C) with similar mean values of temperature and luminance coefficients, Q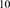 and K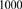, of around 0.7. The effect of the firing precision on the encoding window is remarkable (Table 2), which might explain why its Q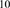 weakly depends on luminance. Similarly, the response latency reduces with light or temperature (Fig. 3D). There is a big difference in the amplitude of the temperature and luminance coefficients: whereas the mean Q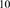 is about 0.8, the mean K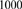 is 0.33. The correlations between the latency and the mean firing rate and precision are similar (Table 2), which suggests that the influence of the firing precision on the temporal scale of the system is as important as the impact of the mean firing rate.

### Modeling

**Figure 4:**
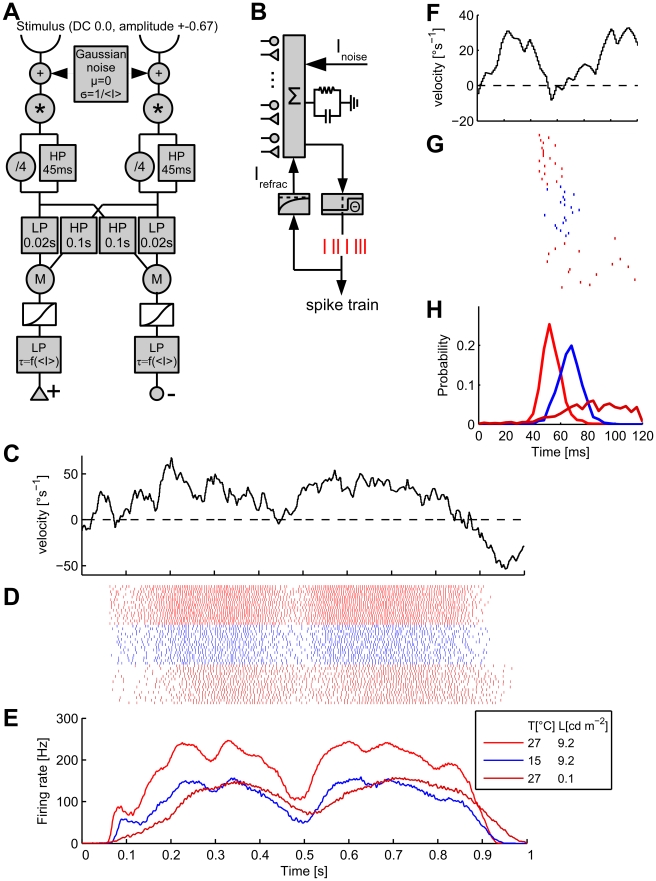
Model structure and simulation results. **A** Structure of the elementary motion detection circuit of the model. **B** Schematic of the Integrate-and-Fire neuron used for simulating H1 responses. **C** A segment of the image velocity as a function of time, same excerpt as shown in Fig. 1A. **D** Simulated raster plot and **E** Simulated average firing rates for each out of three stimulus conditions (averaged over 1500 trials) as indicated in the legend. **F** Segment of a stimulus waveform during a velocity transition from inhibitory to excitatory direction; same excerpt as shown in Fig. 2D. **G** Simulated raster plots of the responses in 20 trials at three different conditions. **H** Probability distribution of the arrival time of the first spike for the three different conditions shown above. Legend as in 4E.

In order to gain insight how temperature and luminance affect the motion processing pathway from the photoreceptors up to H1, we implemented a model of the system under study (see Materials and Methods, Modeling). This model (Fig. 4A, B) incorporates temperature and luminance dependent photoreceptor impulse responses taken from [6], an array of elementary motion detectors [34], [35], and an Integrate-and-Fire model cell that spatially integrates over the array of motion detector inputs. Our aim was to reproduce the measured firing rates and firing precision for the three stimulus conditions depicted in Fig. 1C and Fig. 2D. In particular, the model should be able to reproduce the temperature dependent effects - change of response strength, conservation of firing precision - and the luminance dependent effects - changes of both firing rate and firing precision. The model modifications necessary for reproducing the measurements depicted in Fig. 1C and Fig. 2D thus should allow us to hypothesize what parameters of the motion processing pathway are influenced by the two different sources of perturbation, temperature and luminance, and in which way. The results of the simulation are shown in Fig. 4D, E, G, H.

We first fit the model to reproduce the results for the bright-warm stimulus condition (red traces in Fig. 1, 2). We then went on to modify as few parameters as possible to achieve proper fits for the bright-cold (blue traces) and dark-warm (light red traces) stimulus conditions. Our first finding is that changes at the output level, i. e. the Integrate-and-Fire neuron, alone are able to explain both the reduction in firing rate (cf. Fig. 1C, 4E) and the conservation of firing precision (cf. Fig 2D, 4H) under bright-cold conditions. To this end, we increased the time constant of an exponentially decaying outward current (called refractory current) following each spike. We could also reproduce the reduction of the firing rate by decreasing the output cell's sensitivity to synaptic input by increasing the threshold value 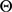 for spike initiation. However, this modification also significantly reduced the firing precision of the cell. We, therefore, suggest that the observed temperature-dependent characteristics of the measured responses may be due solely to a modification of the spiking mechanism (e. g., changes in the kinetic rates of voltage-dependent ion channels), as opposed to a decrease of the synaptic input.

The response of H1 under dark-warm conditions, when compared to the bright-warm condition, is characterized by a decrease of the firing rate, a loss of temporal precision and an increased response latency. Based on the assumption that H1's biophysical properties remained unchanged during reduced brightness, we aimed to reproduce these effects by parameter changes in the motion detection pathway presynaptic to the H1 model. It is tempting to explain the change in response dynamics by known adaptations to low luminance in both photoreceptors [6] and their postsynaptic partners, the large monopolar cells (LMC) [36] of the lamina. Under these conditions, both cells increase their time constant leading to an increased low-pass filtering of the input signal. However, we found that these adaptations cannot explain the virtual absence of short term velocity modulations in the response under dark-warm conditions. From the viewpoint of a photoreceptor, different velocities of a periodic grating are encoded as membrane potential oscillations with varying frequencies set by the stimulus velocity. Applying a temporal filter to the photoreceptor input therefore merely affects how different frequencies (i. e., stimulus velocities) are attenuated, but does not influence how well the motion detector output follows the change in stimulus velocities.

Given the maximum stimulus velocity of about 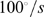 and the spatial wavelength of 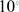, the maximum temporal frequency encoded by the photoreceptors is about 10Hz, which is way below the cut-off frequency of photoreceptors and LMCs under all conditions. An increased filter time-constant in the input lines to the motion detector can therefore not account for the temporally smeared out time-course of the H1 response under low light levels. Rather, these response characteristics suggest a filtering of the motion-sensitive signal, i. e. after the multiplicative interaction within the motion detector. We accounted for these observations by incorporating a low-pass filter with a luminance-dependent time constant at the output of the motion detection circuit. The decrease in firing precision (cf. Fig 2D, 4H) is both due to a roughly hundred-fold increase of noise at the photoreceptors as well as the final low-pass filter. The decrease of the firing rate for dark stimuli is likely a consequence from smaller photoreceptor and LMC responses to dark stimuli as measured previously [36]. We incorporated this effect into our model by scaling down the photoreceptor impulse response for dark stimuli.

The model, as depicted in Fig. 4A, is unable to reproduce the increase in the response latency for dark stimuli, in spite of using photoreceptor impulse responses measured under such circumstances [6]. In order to fit the model parameters and produce the results shown in Fig. 4E, H, we therefore manually introduced a delay of 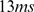 as found by cross-correlation of the measurements with the simulated response.

### Final Conclusions

**Figure 5:**
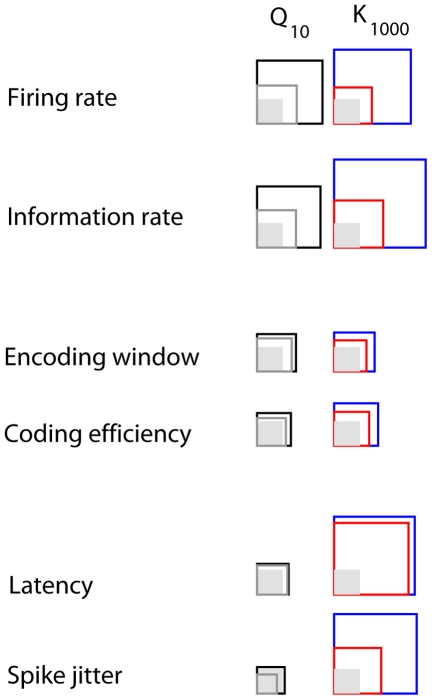
Overview of the results. The mean coefficient of temperature (Q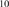) for luminances up to 1 cd m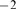 are represented by black squares, and for luminances above 1 cd m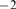, by gray squares. The shaded region correspond to coefficient of 1. Coefficients smaller than 1 were inverted, for sake of comparison. Similarly, the blue squares represent the mean amplitudes of the coefficient of luminance (K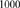) for temperatures up to 22

In summary, the various response parameters can be grouped into three different classes according to whether or not they are affected by temperature and/or luminance (Fig. 5). The firing rate and the information rate are influenced by both temperature and luminance. The encoding window and the coding efficiency are barely affected by temperature and by luminance. Finally, the latency and spike jitter are mainly affected by luminance, but only weakly if at all by temperature.

C, whereas red squares represent the mean amplitudes for higher temperatures.

Our model could reproduce the decrease in firing rate and conservation of spiking precision for cold conditions by incorporating a refractory current following each spike with a temperature-dependent time constant. The decrease in temporal precision for dark stimuli was modeled by adding a higher amount of noise to the photoreceptor input and lowpass-filtering the motion-detector output with a luminance-dependent time constant. The decrease in firing rate for darker stimuli is likely a consequence of reduced photoreceptor response amplitudes and was modeled by a lowering the photoreceptor impulse response.

The near independence of latency and spike jitter from temperature is in contrast to the latency of photoreceptor responses for flash stimuli, which have Q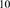 values between 0.35 and 0.66 [6], [7]. Thus, it seems that the latency of the system depends less on temperature than the latency of the photoreceptors. Together with the remarkable correlation between H1's latency and firing precision, and with the fact that responses after velocity transitions are more delayed than in steady firing, this suggests that photoreceptors are not the bottleneck of the latency in the system: considerable amount of delay is introduced in the response during motion computation and possibly during H1's dendritic integration. This is in agreement with our model simulations, which required suitable parameter changes in later stages to mimick the experimental results. Moreover, faster responses in the first stage of the visual system do not improve firing precision in subsequent stages. The response property which seems to be used to control the information throughput when perturbations arise is the mean firing rate. This dominance of the mean firing relative to the firing precision is less pronounced in other sensory modalities like audition [36], but has also been observed in other systems, like in the retina of guinea pigs [37] or in the proprioceptive afferents in crustacean limbs [39].

## Materials and Methods

### Preparation, Recording and Temperature Control

*Calliphora vicina* flies were maintained in the department stock at 19–22

C, 50–60% relative humidity and 12h-12h light-dark cycle. We recorded from 84 female flies, 7–14 days after eclosion. After immobilization with wax, a small hole was cut in the back of the head and the air sacs and fat tissues that cover the lobula plate were put aside. The fly was then transferred into a metallic case which enclosed its entire body except the head. To isolate the head thermally from the environment a soft airstream was blown frontally on it. Ringer's solution was added regularly to the brain to prevent dehydration. The temperature of the case, airstream and solution was regulated by a controlled Peltier device. Head temperature was measured using a microthermoprobe (AD instruments) and a thermometer (GMH3210, Greisinger electronics, Germany). Since the microthermoproble caused considerable tissue damage, temperature and electrophysiological recordings could not be done simultaneously. The head temperature was therefore inferred from the temperature of the metallic support. The relation between head and support temperatures was measured in pilot acquisitions for three different flies. Tungsten electrodes (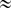
1M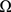) were used for recordings from the H1 neuron, which was identified by its position in the optic lobe and its characteristic response to visual stimuli. After amplification and filtering, H1 responses were processed on-line by a threshold unit which generated a pulse of 1.2 ms when a spike was detected. The pulses were then sampled at 1kHz and saved in a microcomputer for off-line analysis.

### Visual Stimulation

The visual stimulus was presented on CRT-monitor (M21LMAX, Image systems corp., USA) updated at 240 Hz. The visual field stimulation has a area of 72

×83, starting at head midline (azimuth = 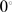). The image used was a square-wave grating with 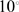 spatial wavelength and contrast of 67%. The image velocity 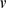 was drawn from a Gauss-Markov process [40], with mean of 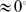 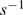, standard deviation of 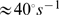 and correlation time of 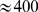
ms. For each acquisition, the stimulus, whose duration was ten seconds, was repeated 150 times, with a interval of two seconds between trials. The image was presented without motion for two minutes before the start of the acquisition, to adapt the photoreceptors to the mean luminance level. The first fifteen trials and the first second of each trial were discarded to avoid accommodation effects and transient responses.

### Data Analysis

The response stationarity over trials was quantified by an accommodation index, defined as the ratio of spike count of the first and hundredth trials. Since a higher accommodation rate would overestimate the response variability, only acquisitions with accommodation indexes between 0.7 and 1.3 (338 from 359 acquisitions) were used for further analysis. These limits were determined based on an analysis of a surrogate data, with several different accommodation indexes. We estimate that a reduction of 0.3 in the index represents a reduction of 5% in the information rate, in relation to an acquisition with the same mean firing rate and approximately unitary index. The estimation of the response variability might also be compromised if the responses entrain with the refresh rate of the video monitor. The degree of entrainment was measured by the residual power of the mean time-dependent firing rate at 238–242Hz. Power spectra were calculated using the Welch-Bartlett method [41], with sampling frequency of 1KHz, window length 512 ms, with an overlap of 256ms. Other window lengths and overlaps yielded similar results. The residual power was obtained by removing linear trends in the spectra. Acquisitions in which the averaged residual power within the interval 238–242 Hz was higher than 0.37 dB - three standard deviations of the residual power distribution for all acquisitions - were discarded (13 out of 359 acquisitions).

To calculate the information rate between stimulus and response, spike trains were discretized in bins of 2ms and the probability distribution of binary words 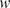 of length 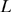 was estimated. The information rate was then calculated as
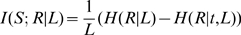
where 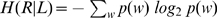 is the entropy of the response, and 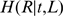 is the entropy of the response given a particular time 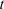 relative to the stimulus, averaged over 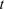. The coding efficiency was then calculated as
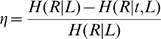
The optimal encoding window was defined as the word length in which the information rate was maximal
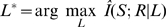
Similarly, the response latency was estimated as the lag 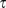 between image velocity and response that maximized 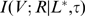, that is
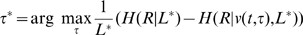
To calculate 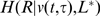, the image velocity 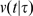 was discretized in bins of 1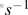.

To reduce the bias of the entropy estimations, a combination of jackknife [42] and shuffled response surrogates was applied. The corrected information rate was calculated as [43]

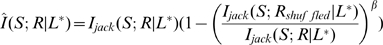
where 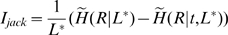. The jackknife estimator 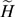 was calculated as
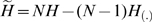
where
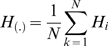
and 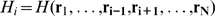 is the estimation of the entropy 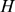 without using the trial 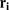 and 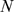 is the total number of trials. A pilot acquisition with 596 trials was used to determine the estimation error as a function of the number of trials. The 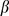 that minimizes the mean bias for 137 trials was 1.13. The maximal difference between the information estimations using 100 and 596 trials was around 1%, for a binwidth 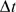 of 2ms and word lengths 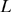 between 2 and 20ms.

The contribution of the precise spike times to the information rate at the optimal encoding windows was determined by a comparison with a second encoding mode, in which only the number of spikes within the word was considered to discriminate different words.

Statistical significance was assessed by non-parametric statistical tests - Wilcoxon sign rank for single, Wilcoxon rank sum for double and Kruskal-Wallis for multiple comparisons. All tests were two-tailed. The sample size for each condition for double comparison was estimated as 8 independent measurements (detected difference of 10%, with size of 0.08 and power of 0.85) [44]. Error bars reported in the graphs are confidence intervals (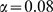), calculated using non-parametric bootstrap with one thousand replications [45]. To measure the correlation between random variables, zero and first order Spearman rank correlation coefficients were used.

The temperature coefficient 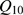 of a determined response property 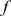 was defined as
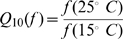
where 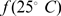 and 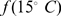 were obtained from a linear least-square fit of the data. The coefficient of luminance 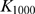 was defined as
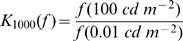
and calculated in the same way as 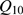.

### Modeling

Input signals ranging from −0.67 to 0.67 were computed from the velocity profile used for the experiments for an array of 80 photoreceptors spaced at 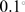 spatial resolution. We added Gaussian noise to each of these input signals (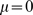, 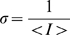, with 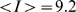 or 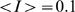) and convolved them with photoreceptor impulse responses taken from [6]. The photoreceptor output was fed through a further filter stage consisting of a high-pass filter (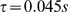) and 25% of the unfiltered photoreceptor output. 60 pairs of photoreceptor outputs with a spatial distance of 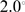 were processed by an array of so-called Reichardt-Detectors [35]. One such Reichardt Detector consisted of two mirror-symmetrical subunits, each multiplying the low-pass-filtered signal (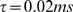) of one input line by the high-pass-filtered signal (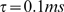) of the neighboring input line. The output signals of the detectors were further processed by a sigmoidal synaptic non-linearity (
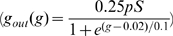
) and a low-pass-filter (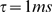 for bright stimuli, 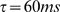 for dark stimuli) before providing excitatory and inhibitory input to the output model neuron. The final stage was an Integrate-and-Fire neuron (Fig. 4B) integrating over all synaptic inputs. It also received a noise current (Gaussian distributed, 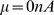, 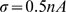) and a refractory current following each spike (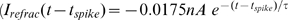, with 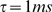 for the warm and 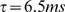 for the cold condition). Membrane currents are described by 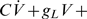 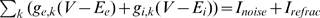, with 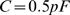, 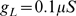, 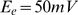, 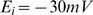, and 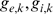 the outputs of the 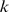-th motion detector. This ordinary differential equation was simulated using the explicit Euler method. A spike was fired when the potential 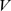 crossed a threshold of 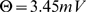 above the resting potential. The firing rates depicted in Fig. 4E and the distribution of the first spikes after a velocity transition in Fig. 4H were computed by averaging over 1500 trials.

The parameters of the model were fit manually to minimize the distance between the measured firing rates and those produced by simulating the model for several hundred trials. We also used genetic algorithms to search for parameter sets that minimize this error but could not improve the results found before during manual search.

## Supporting Information

Figure S1 Information rate as a function of the encoding window. a. The information rate between stimulus and a representative H1 response as a function of the encoding window, for timing and count encoding modes. Lr* is the encoding window where the information rate in the timing mode is maximal. b. Respective coding efficiencies. c. Information rates (timing mode) between the discretized velocity and the response, calculated at the optimal encoding window. Response latency was defined as the lag that maximizes the information rate.
(0.08 MB PDF)

## References

[1] Heinrich B (1993). The Hot-blooded Insects: Strategies and Mechanisms of Thermoregulation.

[2] Casey TM (1992). Biophysical ecology and heat exchange in insects.. Am Zool.

[3] May ML (1979). Insect thermoregulation.. Annu Rev Entomol.

[4] Stavenga DG, Schwering PBW, Tinbergen J (1993). A three-compartment model describing temperature changes in tethered flying bowflies.. J Exp Biol.

[5] Hille B (2001). Ion Channels of Excitable Membranes.

[6] Roebroek JGH, van Tjonger M, Stavenga DG (1990). Temperature dependence of receptor potential and noise in fly (*Calliphora erythrocephala*) photoreceptor cells.. J Insect Physiol.

[7] Tatler B, O'Carroll DC, Laughlin SB (2000). Temperature and the temporal resolving power of fly photoreceptors.. J Comp Physiol [A].

[8] Hengstenberg R (1971). Das Augenmuskelsystem der Stubenfliege *Musca domestica* 1. Analyse der “clock-spikes” und ihrer Quellen.. Kybernetik.

[9] Warzecha AK, Horstmann W, Egelhaaf M (1999). Temperature-dependence of neuronal performance in the motion pathway of the bowfly *Calliphora erythrocephala*.. J Exp Biol.

[10] Faucherre J, Cherix D, Wyss C (1999). Behavior of *Calliphora vicina* (Diptera, Calliphoridae) under extreme conditions.. J Insect Behav.

[11] El-Wadawi R, Bowler K (1995). The development of thermotolerance protects blowfly flight muscle mitochondrial function from heat damage.. J Exp Biol.

[12] Laughlin SB, Howard J, Blakeslee B (1987). Synaptic limitation to contrast coding in the retina of the blowfly *Calliphora*.. Proc R Soc Lond B.

[13] Borst A, Haag J (2001). Effects of mean firing on neural information rate.. J Comput Neurosci.

[14] Lewen G, Bialek W, de Ruyter van Steveninck RR (2001). Neural code of naturalistic motion stimuli.. Network: Comput Neural Syst.

[15] Egelhaaf M, Grewe J, Kern R, Warzecha AK (2001). Outdoor performance of a motion-sensitive neuron in the blowfly.. Vision Res.

[16] Grewe J, Kretzberg J, Warzecha AK, Egelhaaf M (2003). Impact of photon noise on the reliability of a motion sensitive neuron in the fly's visual system.. J Neurosci.

[17] Borst A (2003). Noise, not stimulus entropy, determines neural information rate.. J Comput Neurosci.

[18] Hausen K, All M (1984). The lobula-complex of the fly: structure, function and significance in visual behaviour.. Photoreception and vision in invertebrates.

[19] Strausfeld N, Ali MA (1984). Functional neuroanatomy of the blowfly's visual system.. Photoreception and Vision in Invertebrates.

[20] Borst A, Haag J (2002). Neural networks in the cockpit of the fly.. J Comp Physiol [A].

[21] Haag J, Borst A (2001). Recurrent network interactions underlying flow-field selectivity of visual interneurons.. J Neurosci.

[22] Haag J, Borst A (2002). Dendro-dendritic interactions between motion-sensitive large-field neurons in the fly.. J Neurosci.

[23] Haag J, Borst A (2003). Orientation tuning of motion-sensitive neurons shaped by vertical-horizontal network interactions.. J Comp Physiol.

[24] Farrow K, Haag J, Borst A (2003). Input organization of multifunctional motion sensitive neurons in the blowfly.. J Neurosci.

[25] Farrow K, Haag J, Borst A (2006). Nonlinear, binocular interactions underlying flow field selectivity of a motion-sensitive neuron.. Nature Neurosci.

[26] Gronenberg W, Strausfeld NJ (1990). Descending neurons supplying the neck and flight motor of diptera: physiological and anatomical characteristics.. J Comp Neurol.

[27] Hengstenberg R (1991). Gaze control in the blowfly *Calliphora*: a multisensory, two-stage integration process.. Neurosciences.

[28] Haag J, Wertz A, Borst A (2007). Integration of lobula plate output signals by dnovs1, an identified premotor descending neuron.. J Neurosci.

[29] Wertz A, Borst A, Haag J (2008). Nonlinear integration of binocular optic flow by dnovs2, a descending neuron of the fly.. J Neurosci.

[30] Wertz A, Gaub B, Plett J, Haag J, Borst A (2009). Robust coding of ego-motion in descending neurons of the fly.. J Neurosci.

[31] Wertz A, Haag J, Borst A (2009). Local and global motion preferences in descending neurons of the fly.. J Comp Physiol.

[32] Berry-II M, Warland D, Meister M (1998). Refractoriness and neural precision.. J Neurosci.

[33] Land MF, Collett TS (1974). Chasing behavior of houseflies (*Fannia canicularis*) a. description and analysis.. J Comp Physiol.

[34] Hassenstein B, Reichardt W (1956). Systemtheoretische analyse der zeit-, reihenfolgen- und vorzeichenauswertung bei der bewegungsperzeption des rüsselkäfers chlorophanus.. Z Naturforsch.

[35] Borst A, Reisenman C, Haag J (2003). Adaptation of response transients in fly motion vision. ii: Model studies.. Vision Research.

[36] Laughlin SB, Howard J, Blakeslee B (1986). Synaptic limitations to contrast coding in the retina of the blowfly calliphora.. Proc R SocLond.

[37] Rokem A, Watzl S, Gollisch T, Stemmler M, Herz AVM (2006). Spike-timing precision underlies the coding efficiency of auditory receptor neurons.. J Neurophysiol.

[38] Koch K, McLean J, Segev R, Freed MA, Berry-II MJ (2006). How much the eye tells the brain.. Current Biology.

[39] DiCaprio R, Billimoria C, Ludwar B (2007). Information rate and spike-timing precision of proprioceptive afferents.. J Neurophysiol.

[40] Bartosch L (2001). Generation of colored noise.. Int J Mod Phys C.

[41] Manolakis DG, Ingle VK, Kogon SM (2005). Statistical and adaptive signal processing.

[42] Quenouille MH (1956). Notes on bias in estimation.. Biometrika.

[43] Optican LM, Gawne TJ, Richmond BJ, Joseph PJ (1991). Unbiased measures of transmitted information and channel capacity from multivariate neuronal data.. Biol Cybern.

[44] Gibbons J, Chakraborti S (2003). Nonparametric statistical inference, fourth edition.

[45] Zoubir AM, Boashash B (1998). The bootstrap and its application in signal processing.. IEEE Signal Proc Mag.

