# Supplementary material for: Coding Efficiency of Fly Motion Processing Is Set by Firing Rate, Not Firing Precision
Source: PLoS Comput Biol. 2010 Jul 22;6(7):e1000860. doi: 10.1371/journal.pcbi.1000860 (PMC2908696; doi:10.1371/journal.pcbi.1000860)
Supplement: Figure S1 — Information rate as a function of the encoding window. a. The information rate between stimulus and a representative H1 response as a function of the encoding window, for timing and count encoding modes. Lr* is the encoding window where the information rate in the timing mode is maximal. b. Respective coding efficiencies. c. Information rates (timing mode) between the discretized velocity and the response, calculated at the optimal encoding window. Response latency was defined as the lag that maximizes the information rate. (0.08 MB PDF) [file pcbi.1000860.s001.pdf]

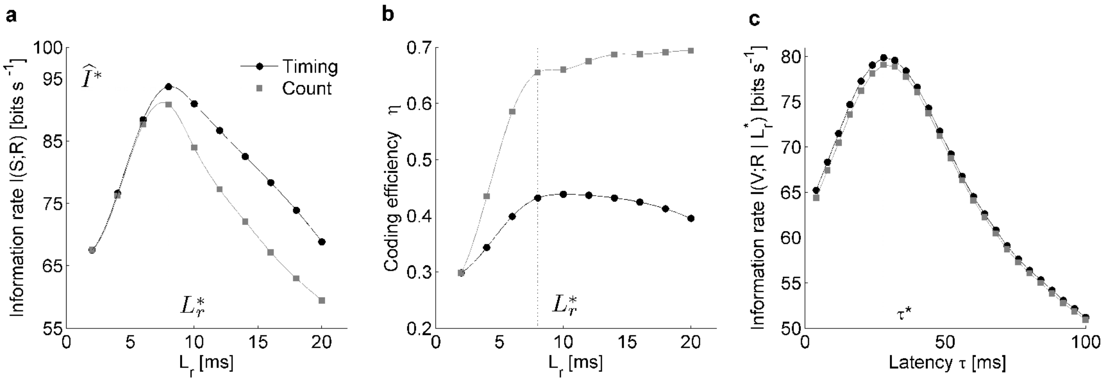

Figure S1: Information rate as a function of the encoding window. **a.** The information rate between stimulus and a representative H1 response as a function of the encoding window, for timing and count encoding modes. Lr* is the encoding window where the information rate in the timing mode is maximal. **b.** Respective coding efficiencies. **c.** Information rates (timing mode) between the discretized velocity and the response, calculated at the optimal encoding window. Response latency was defined as the lag that maximizes the information rate.
